# Cladistic Relationships and Landscape Genetics of the Endangered Indian Peacock Softshell Turtle *Nilssonia hurum* (Gray, 1830): Implications for Strategic Conservation Planning

**DOI:** 10.1002/ece3.72751

**Published:** 2025-12-22

**Authors:** Imon Abedin, Angkasa Putra, Hye‐Eun Kang, Arunima Singh, Shailendra Singh, Hilloljyoti Singha, Hyun‐Woo Kim, Shantanu Kundu

**Affiliations:** ^1^ Wildlife Ecology Lab, Department of Zoology Bodoland University Kokrajhar India; ^2^ Interdisciplinary Program of Marine and Fisheries Sciences and Convergent Technology Pukyong National University Busan Republic of Korea; ^3^ Institute of Marine Life Science Pukyong National University Busan Republic of Korea; ^4^ Turtle Survival Alliance Foundation India Lucknow India; ^5^ Centre for Wildlife Research and Biodiversity Conservation Bodoland University Kokrajhar India; ^6^ Department of Marine Biology, College of Fisheries Science Pukyong National University Busan Republic of Korea; ^7^ Research Center for Marine Integrated Bionics Technology Pukyong National University Busan Republic of Korea; ^8^ Marine Integrated Biomedical Technology Center, National Key Research Institutes in Universities, Pukyong National University Busan Republic of Korea; ^9^ Ocean and Fisheries Development International Cooperation Institute, College of Fisheries Science Pukyong National University Busan Republic of Korea; ^10^ International Graduate Program of Fisheries Science Pukyong National University Busan Republic of Korea

**Keywords:** climate change, ecology, mitogenome, phylogeny, species distribution model, testudines

## Abstract

The endangered Peacock Softshell Turtle *Nilssonia hurum* (Gray, 1830) has undergone a steep population decline in recent decades because of habitat loss and anthropogenic pressures, highlighting the urgent need for scientific intervention to ensure its protection in the wild. Thus, the present study integrates mitogenomic and ecological data to guide proactive conservation strategies for this species. The study reports the first mitogenome (16,788 bp) of *N. hurum* from the upper Ganges region, which exhibits a typical gene composition and strong A + T bias. The mitogenome‐based phylogenetic analyses reveal the monophyly of the genus *Nilssonia* Gray, 1872 and a close evolutionary relationship between *N. hurum* and *N. nigricans* (Anderson, 1875). The genetic distance and haplotype network analyses on the basis of the *CYTB* gene reveal substantial intraspecific diversity and spatial genetic structuring among populations across river basins within the easternmost range. Using species distribution modeling, the study identified 123,699 km^2^ (6.81% of IUCN range) as presently suitable for *N. hurum* . However, future climate projections indicate drastic reductions in suitable habitat, with losses of up to 85% due to climate change. The landscape genetic analyses revealed that the Meghna basin exhibits the highest mean functional connectivity (0.603), whereas the Brahmaputra basin shows the lowest connectivity (0.198) despite containing suitable habitat patches, consistent with its high genetic diversity. Moreover, projections under future climate scenarios, driven by anticipated losses in habitat suitability, indicate widespread declines in functional connectivity across all basins and sub‐basins. The landscape geometry assessments further reveal increasing habitat fragmentation due to climate change. Therefore, populations persisting within suitable habitat patches across different river basins in the eastern range should be prioritized as distinct conservation units for future management. Overall, this study provides a critical foundation for site‐specific conservation planning through landscape genetics to address habitat loss, fragmentation, and mitigating inbreeding depression for ensuring the long‐term endurance of this threatened freshwater turtle in South Asia.

## Graphical abstract

This study provides the first mitogenome of the endangered Peacock Softshell Turtle ( *Nilssonia hurum* ), revealing its evolutionary relationships and moderate genetic diversity across river basins. Species distribution modeling predicts up to 85% habitat loss under future climate scenarios, alongside severe declines in functional connectivity and rising fragmentation through landscape genetic approach. The findings emphasize prioritizing basin‐specific populations as distinct conservation units to guide effective conservation planning.

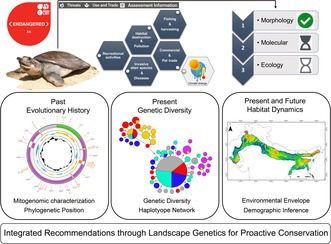

## 1 Introduction

Over recent decades, conservation science has undergone a paradigm shift, increasingly adopting interdisciplinary approaches to improve the effectiveness of species protection efforts (Zenboudji et al. 2016). This transition has been largely accelerated by the rapid and ongoing loss of biodiversity, coupled with the emergence of novel analytical and technological tools that allow for a more holistic understanding and management of any threatened taxa (Rissler 2016; Weiskopf et al. 2024). More crucially, climate change is regarded as one of the primary drivers of global biodiversity decline because of its profound influence on environmental regimes and its role in accelerating species turnover (Hewitt 2004; Potter et al. 2018). These climatic shifts and biome configurations have driven extensive reshaping of geographic ranges of species across different zoogeographic regions (Wiens and Donoghue 2004; Hernandez Fernandez and Vrba 2005; Jetz and Fine 2012; Landis et al. 2021; Gamboa et al. 2024). Concurrently, the global freshwater crisis continues to intensify, with many ecosystems facing accelerated degradation and loss (Reid et al. 2019). This decline is particularly alarming, given the disproportionate biodiversity harbored by freshwater habitats (Sayer et al. 2025). Among the most impacted taxa are freshwater turtles (order Testudines), which represent an ancient reptilian lineage with a fossil record exceeding 200 million years (Thomson et al. 2021). These species play crucial ecological roles, including nutrient cycling and food web regulation (Santori et al. 2020). Yet, they are experiencing unprecedented population declines because of synergistic threats such as habitat loss, overexploitation, and climate change (Butler 2019; Stanford et al. 2020; Willey et al. 2022).

Despite their ecological importance, the conservation of freshwater turtles has often been insufficiently informed by integrative scientific frameworks (Tilman et al. 2017; Harfoot et al. 2021). Within this context, the family Trionychidae, comprising 35 recognized species under 13 genera, includes several large‐bodied freshwater turtles of conservation concern. The genus *Nilssonia* Gray, 1872 currently represents five extant species, viz., *N. formosa* (Gray, 1869), *N. gangetica* (Cuvier, 1825), *N. hurum* (Gray, 1830), *N. leithii* (Gray, 1872), and *N. nigricans* (Anderson, 1875) (TTWG 2025). Historically regarded as monotypic, *Nilssonia* was expanded following taxonomic revisions that integrated several *Aspideretes* congeners on the basis of morphological and molecular evidence (Engstrom et al. 2004; Praschag et al. 2007). These species are primarily distributed across South and Southeast Asia and are known to fulfill critical roles as scavengers and predators in aquatic ecosystems. However, all five species are currently threatened with extinction, as recognized by the International Union for Conservation of Nature (IUCN) Red List assessment (IUCN 2025).

Among them, the Peacock Softshell Turtle ( *N. hurum* ) is a particularly vulnerable species inhabiting the Indian subcontinent. Originally described as *Trionyx hurum* , it was subsequently reassigned to *Aspideretes* and then to *Nilssonia* following taxonomic refinements (Praschag et al. 2007). Once locally abundant, *N. hurum* populations have experienced sharp declines over recent decades because of extensive harvesting for bushmeat, pet trade, and traditional medicine markets, with trade volumes reportedly reaching up to 80 tons per week in the late 1990s (Choudhury and Bhupathy 1993; Das et al. 2021). Concurrently, habitat degradation is another major factor that further exacerbates population declines of this species. As a result, *N. hurum* is currently listed as “Endangered” by the IUCN and included in ‘Appendix I’ of Convention on International Trade in Endangered Species of Wild Fauna and Flora, which prohibits all international commercial trade. In India, it receives the highest level of legal protection under ‘Schedule I’ of the Wildlife (Protection) Amendment Act, 2022. Notably, conservation efforts for *N. hurum* have been severely constrained by the lack of integrated knowledge on its population genetic structure and habitat requirements. Thus, addressing these knowledge gaps necessitates a synergistic approach that combines systematics, genomics, and ecological modeling to inform targeted management strategies (Knowles et al. 2007; Coelho et al. 2024). Such advances enable the selection of critical habitats and colligated populations across its distributional extent, ensuring strategic management and conservation interventions (Safaei et al. 2021; Abedin et al. 2025).

In addition to morphological studies, genetic research on this species has been conducted but has predominantly relied on partial mitochondrial or nuclear loci sequences (Engstrom et al. 2004; Praschag et al. 2007; Liebing et al. 2012; Kundu et al. 2015; Thomson et al. 2021; Yadav et al. 2021). These preliminary analyses have offered initial insights into its genetic diversity, phylogenetic relationship, and taxonomic placement within trionychids. Nevertheless, advancements in genomic studies have underscored the importance of the complete mitogenome in elucidating more precise phylogenetic interpretation (Zardoya and Meyer 1998; Kumazawa and Nishida 1999; Mindell et al. 1999). Globally, complete mitogenomes have been sequenced for numerous freshwater turtles, thereby improving understanding of their systematics and evolutionary history (Kundu et al. 2018; Kundu et al. 2023). However, the complete mitochondrial genome of *N. hurum* remains uncharacterized, constraining our ability to infer its matrilineal evolutionary trajectory within the broad lineage of Testudines.

Beyond morphological and genetic approaches, it is also imperative to understand the habitat dynamics of this threatened species to develop integrated management strategies under changing climatic conditions (Sommer et al. 2013). Furthermore, integrating genetic data with Species Distribution Models (SDMs) offers a holistic framework for understanding spatial genetic dynamics, thereby enhancing the effectiveness of more accurate conservation initiatives (Claerhout et al. 2023; Kundu et al. 2023; Abedin et al. 2025). The integration regarded as landscape genetics has shown considerable promise in providing robust approaches to understanding how landscape features shape realized population connectivity across a region (Spear et al. 2005; Cushman et al. 2006; Storfer et al. 2010; Balkenhol et al. 2009; Mateo‐Sánchez et al. 2015). This approach, which integrates pairwise genetic distances and pairwise effective distances derived from environmental data, has shown considerable potential for guiding proactive conservation strategies (Gutiérrez‐Rodríguez et al. 2017; Huang et al. 2024; Ajene et al. 2024).

**FIGURE 1:**
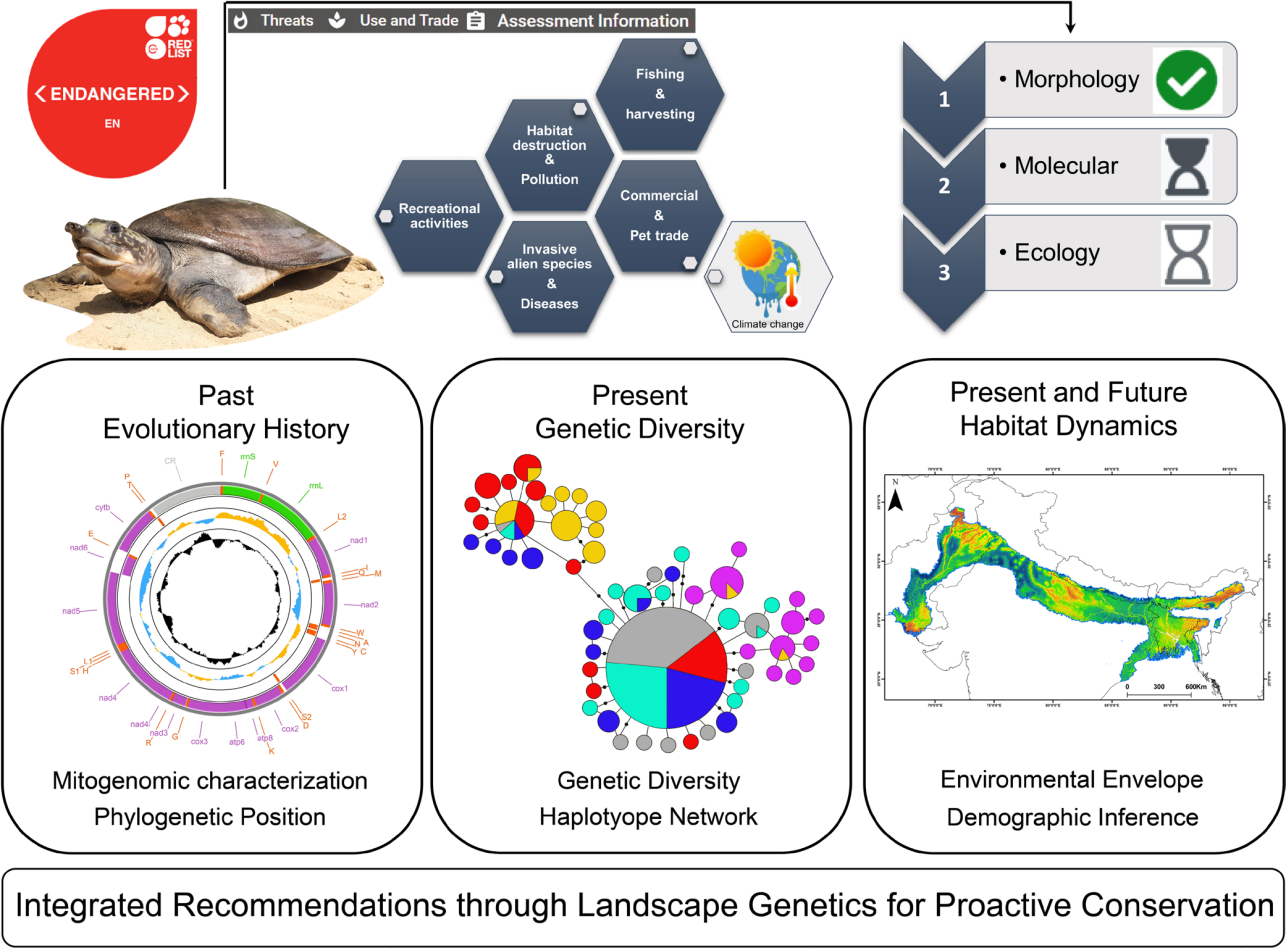
The integrated workflow employed in this study depicts the evolutionary history, genetic diversity, and habitat dynamics of *Nilssonia hurum* across South Asia, providing robust landscape genetic insights to support evidence‐based conservation strategies. All representative figures and maps were generated by the authors, and the species photograph was provided by Sreeparna Dutta (TSAFI).

Accordingly, this study employs an integrative framework to bridge current knowledge gaps and guide the development of proactive conservation strategies for this imperiled species, with the following specific objectives: (i) sequencing the novel mitogenome of *N. hurum* through a next‐generation sequencing approach for characterization and elucidating the matrilineal evolutionary relationship within the broader Testudines lineage, (ii) conducting CYTB‐gene analyses to examine population genetic structure, (iii) identifying suitable habitats for the species under present and future climatic scenarios, and (iv) integrating genetic data with habitat suitability within a landscape genetics framework to elucidate functional corridor connectivity for the species under current and projected environmental conditions. Thus, by implementing this unified approach, the study provides novel genomic resources and identifies climatic refugia as well as functional corridors for evidence‐based conservation planning to ensure the long‐term survival of *N. hurum* (Figure 1).

## 2 Materials and Methods

### 2.1 Biological Sampling and Ethics Statement

A single specimen of *N. hurum* was sourced from the species colony maintained by the Turtle Survival Alliance Foundation India (TSAFI) in Uttar Pradesh, India. The identification of species was confirmed on the basis of taxonomic characters as per the previous literature (Das et al. 2010). All essential permits for sample collection were procured from the Principal Chief Conservator of Forests (Wildlife), Lucknow, Uttar Pradesh, India permit number 1451/23–2‐12 (G), provided by the Department of Environment, Forest, and Climate Change, Government of Uttar Pradesh, India. The blood sample (500 μL) was taken from the posterior limb after Alfaxalone sedation (20–30 mg/kg) and preserved in a 1.5 mL EDTA‐coated 2 mL centrifuge tube and preserved at 4°C. No animals were harmed, sacrificed, or retained during this research. The sampled specimen was promptly returned to its natural environment after biological sample collection. All protocols followed the ethical standards outlined in the ARRIVE 2.0 guidelines (https://arriveguidelines.org) for animal research (Percie du Sert et al. 2020).

### 2.2 Mitogenome Extraction and Next‐Generation Sequencing

Total genomic DNA was extracted using the Alexgen DNA Kit (Alexius Biosciences, Ahmedabad, Gujarat, India) following the manufacturer's protocols (Ahmad et al. 2007). The genomic DNA concentration and quality were assessed employing a Qubit 4.0 fluorometer (Thermo Fisher Scientific, USA). The library preparation and mitogenome sequencing were performed at Unipath Specialty Laboratory Ltd. (http://www.unipath.in/), Ahmedabad, India. A paired‐end sequencing library was constructed using the QIAseq FX DNA Library Kit (Cat. No. 180479, Qiagen), with DNA fragmentation achieved via a Covaris M220 Focused Ultrasonicator (Covaris Inc., San Diego, CA, USA), and Illumina‐specific adapters were ligated to both ends of the DNA fragments. The high‐fidelity amplification was carried out using the HiFi PCR Master Mix (Takara Bio Inc., Japan) to improve coverage from low‐input DNA. Library quality and fragment size distribution were evaluated using the TapeStation 4150 system (Agilent Technologies, USA) with High Sensitivity D1000 ScreenTape, according to the manufacturer's protocol. The libraries that passed quality control were sequenced through the Illumina NovaSeq 6000 platform (Illumina, USA) to generate high‐throughput paired‐end reads.

### 2.3 Mitochondrial Genome Assembly and Annotation

Approximately 20 million paired‐end reads were utilized for mitogenome assembly and annotation by using Geneious Prime v2023.0.1 (Kearse et al. 2012). The gene boundaries were confirmed through two annotation pipelines: MITOS on the Galaxy web server (https://usegalaxy.eu) and MitoAnnotator (http://mitofish.aori.u‐tokyo.ac.jp/annotation/input/) (Bernt et al. 2013; Iwasaki et al. 2013). To ensure accurate identification of protein‐coding genes (PCGs), predicted coding sequences were translated into putative amino acid sequences using the standard vertebrate mitochondrial genetic code via the ORF Finder tool (https://www.ncbi.nlm.nih.gov/orffinder/). The curated mitogenome was submitted to GenBank, alongside a fully detailed gene feature file comprising annotation and strand information. A circular mitogenome map of *N. hurum* was generated through MitoAnnotator, with manual verification of intergenic spacers and overlapping regions. The sequence lengths and base compositions of PCGs, ribosomal RNAs (rRNAs), and transfer RNAs (tRNAs) were evaluated in MEGA v12 (Kumar et al. 2024).

### 2.4 Mitogenomic Phylogenetic Analyses

To explore the matrilineal phylogenetic relationship of *N. hurum* within the larger taxonomic framework of the order Testudines, 31 species mitogenomes were acquired from the GenBank database (Table S1). The phylogenetic construction included 29 species across 10 families under the suborder Cryptodira. The representative species mitogenomes within the suborder Pleurodira were fetched from GenBank to serve as outgroup taxa. Additionally, to develop a robust phylogenetic relationship among the targeted taxa, all 13 PCGs were concatenated and aligned using iTaxoTools v0.149 (Vences et al. 2021). The model selection for nucleotide substitution was conducted using PartitionFinder 2, identifying the GTR + G + I model as the most appropriate (Lanfear et al. 2017). The phylogenetic analyses were carried out using both Bayesian Inference (BI) and Maximum‐likelihood (ML) approaches. The BI was implemented in MrBayes v3.1.2, employing the nst = 6 setting and running one cold chain and three heated Metropolis‐coupled Markov Chain Monte Carlo (MCMC) chains for 10 million generations (Ronquist et al. 2012). The tree was performed every 100 generations along with the initial 25% of samples discarded as burn‐in. The MCMC analysis was conducted until convergence was achieved, indicated by a standard deviation of split frequencies reaching 0.01 and the Potential Scale Reduction Factor (PSRF) for all parameters approaching 1.0. In addition, the ML analysis was executed through PhyML v3.0, applying default parameters with 1000 bootstrap supports to assess branch support (Guindon et al. 2010). The resulting phylogenetic trees were visualized and annotated via the Interactive Tree of Life (iTOL) v4 for improved visualization (Letunic and Bork 2021).

### 2.5 Genetic Distance and Haplotype Network

The haplotype network analysis was conducted using partial *CYTB* gene sequences (*n* = 27) derived from both the present mitogenome generated from the upper Ganges region and previously published data from Barak, Brahmaputra, Ganges, GBM Delta system, Gomati, Meghna, and Subarnarekha riverine systems (Engstrom et al. 2004; Praschag et al. 2007; Liebing et al. 2012; Bhaskar and Mohindra 2018; Yadav et al. 2021) (Table S2). The sequence alignment for the dataset was performed using CLUSTAL X (Thompson et al. 1997), and the genetic distances were estimated using the Kimura 2‐Parameter (K2P) model in MEGA12. The measures of genetic diversity, including the number of haplotypes, segregating and parsimony‐informative sites, nucleotide diversity (π), and haplotype diversity (Hd) were computed using DnaSP v6 (Rozas et al. 2017). Additionally, the haplotype network was constructed using the Templeton, Crandall, and Sing (TCS) method implemented in POPART (Clement et al. 2000; Leigh and Bryant 2015).

### 2.6 Presence Record and Model Extent

**FIGURE 2:**
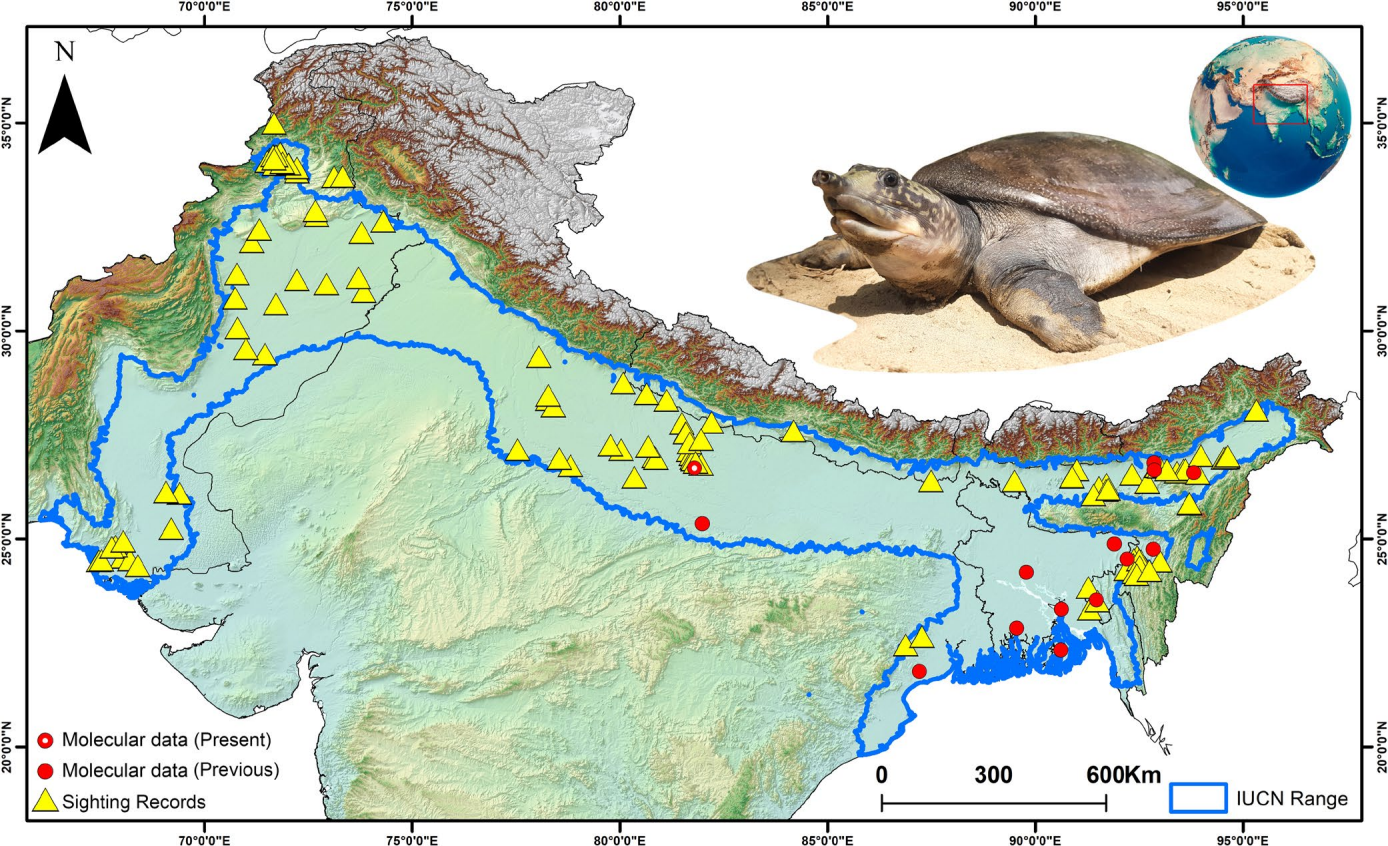
The map illustrates occurrence records and genetic sampling sites compiled from both primary field surveys and secondary data sources within the IUCN‐defined range of the species.

A total of five occurrences of *N. hurum* were sighted by the TSAFI team from distinct drainage systems (Saryu, Mala, Kane, Khanaut, and Gomti) in the Ganges River basin (Figure 2). Additionally, six sightings were confirmed from different localities within the Jia Bharali and Dhansiri Rivers in the Brahmaputra basin. Furthermore, three sightings were also recorded from the Barak River system in Assam state. In addition, five occurrence records were confirmed from wetlands and the Gomati River system in Tripura State in northeast India. Further, to improve the comprehensive representation of presence information, additional secondary occurrence points were accumulated from the IUCN GeoCAT tool, which consolidates species distribution data from several open‐access sources (Bachman et al. 2011). Additional occurrence information was also obtained from published literature and reports (Praschag et al. 2007; Liebing et al. 2012; Kundu et al. 2015; Bhaskar and Mohindra 2018; Yadav et al. 2021; TTWG 2025). In total, 143 presence localities were gathered from both primary and secondary sources, covering the IUCN‐mapped distribution of the species. The records of preserved specimens and captive individuals were filtered out to ensure dataset robustness and minimize sampling bias, as these may not accurately represent true wild occurrence points. Moreover, to minimize spatial autocorrelation and avoid model overfitting, presence points were spatially rarefied at a 1 km^2^ resolution using the SDM Toolbox v2.4 rarefaction tool, aligning with the spatial resolution of the environmental raster layers (Brown et al. 2017). After rarefaction, 125 unique points were selected for model training, and the IUCN‐defined range of *N. hurum* was selected as the model training area. This expert‐validated distribution, provided by the IUCN TFTSG, offers a standardized baseline for SDM studies.

### 2.7 Model Variables for Habitat Suitability

The habitat suitability modeling for *N. hurum* utilized bioclimatic, habitat, anthropogenic, and topographic variables following established SDM protocols (Peterson and Soberón 2012). The standard 19 bioclimatic predictors were acquired from the WorldClim database (Fick and Hijmans 2017). Further, Euclidean distance to water bodies is included as a habitat predictor, derived utilizing the global Land Use Land Cover (LULC) dataset (ESRI Sentinel‐2, 10 m resolution) via the Living Atlas platform (Karra et al. 2021). The categorical dataset was converted and processed into a continuous raster utilizing the Euclidean distance tool in ArcGIS 10.6 (Abedin et al. 2025). The elevation was retrieved from the Shuttle Radar Topography Mission (SRTM) dataset at 90 m resolution (http://srtm.csi.cgiar.org/srtmdata/). Additionally, the Global Human Footprint Dataset was taken as an anthropogenic variable to quantify the Human Influence Index (HII) (Wildlife Conservation Society 2005). The covariate layers were then resampled to 30 arcseconds (~1 km^2^) in ArcGIS v10.6 utilizing the Spatial Analyst extension. The multicollinearity between the variables was tested with the SAHM package in VisTrails, excluding variables with Pearson, Spearman, and Kendall correlation coefficients (*r*) > 0.7 (Warren et al. 2010; Morisette et al. 2013) (Figure S1). Furthermore, the future projections were modeled under Shared Socioeconomic Pathways (SSP) SSP245 and SSP585 for the periods 2041–2060 and 2061–2080. The climate projections were derived from the HadGEM3‐GC31 LL model within the CMIP6 framework (Li et al. 2023; Gautam and Shany 2024).

### 2.8 Ensemble Model Assessment

The ensemble model was applied to estimate habitat suitability using five complementary algorithms: Boosted Regression Trees (BRT), Generalized Linear Models (GLM), Multivariate Adaptive Regression Splines (MARS), Maximum Entropy (MaxEnt), and Random Forests (RF) (Guisan et al. 2007; Elith and Leathwick 2009; Miller 2010). This multi‐algorithmic approach utilized the strengths of individual models to improve prediction accuracy and overall capability (Hao et al. 2020). The model implementation was conducted applying the SAHM (Software for Assisted Habitat Modeling) package in the VisTrails platform (Talbert and Talbert 2012; Morisette et al. 2013). The resultant outputs comprised continuous habitat suitability maps (values 0 = unsuitable to 1 = highly suitable), which were converted into binary presence–absence predictions on the basis of the sensitivity‐equals‐specificity (SES) threshold. A consensus suitability map was generated by combining all five algorithms, with values of pixels ranging from ‘0’ to ‘5’, where ‘5’ indicated complete agreement across models. The performance of the models was assessed using multiple evaluation metrics, including the Area Under the Receiver Operating Characteristic Curve (AUC), True Skill Statistic (TSS), Cohen's Kappa, Proportion Correctly Classified (PCC), sensitivity, and specificity. These metrics were calculated for both the training datasets and via 10‐fold cross‐validation to ensure robustness (Cohen 1968; Allouche et al. 2006; Phillips and Elith 2010; Jiménez‐Valverde et al. 2013).

### 2.9 Landscape Genetics

The genetic distances were interpolated in Alleles in Space v1.0 software, which is based on the partial *CYTB* gene (Miller 2005). The ‘interpolate genetic distance shape’ function was utilized to create a spatial interpolation of genetic distances across the study area, with both the ‘*x*’ and ‘*y*’ coordinate bins set to 100 and the default distance weighting value fixed at 1. This interpolation estimates genetic distances across regions by integrating sequence data with their geographic coordinates. The approach generates a continuous genetic surface map by assigning greater weight to values from spatially proximate points than to those from distant points, thereby revealing genetic structure even in unsampled locations situated nearby. Further, the interpolated data were subsequently imported into ArcGIS v10.6, where a genetic distance raster was produced to visualize spatial patterns and gradients of genetic distance across the assessed extent. The genetic distances were interpolated solely across the eastern range of the species, encompassing multiple river basins (Ganges, Brahmaputra, Subarnarekha, Meghna, Gomati, and Barak) and the Ganges–Brahmaputra–Meghna (GBM) Delta, where genetic data were available and were subsequently used for landscape genetics analyses. This genetic surface was then combined with habitat suitability layers using a multiplicative approach to generate a resistance surface for identification of functional connectivity, following methodologies from previous studies (Mateo‐Sánchez et al. 2015; Mukherjee et al. 2025). Circuit theory, a widely used approach for assessing connectivity, was employed to identify potential corridors within the study area. The corridor simulations were conducted using the Circuitscape toolbox integrated with ArcGIS 10.6 (Wang et al. 2014; McRae et al. 2008). The analyses were performed in pairwise mode, with resistance surfaces rasters derived from the integrated landscape genetics framework.

### 2.10 Assessment of Landscape Shape Geometry

The class‐level landscape metrics were employed to assess the structural and spatial characteristics of suitable habitat patches under both current and projected future conditions. These metrics were computed using FRAGSTATS version 4.2.1, a widely recognized tool in landscape ecology for quantifying spatial patterns and analyzing landscape composition and configuration (McGarigal and Marks 1995; Hesselbarth et al. 2019). The class descriptor tables were derived from habitat suitability outputs corresponding to current and future climate scenarios. The analysis utilized the eight‐cell neighborhood rule and incorporated user‐defined tiles along with a uniform sampling strategy to maintain consistency. The primary landscape metrics included Number of Patches (NP), Patch Density (PD), Total Edge (TE), Largest Patch Index (LPI), Aggregation Index (AI), and Landscape Shape Index (LSI). The NP represents the total number of discrete patches of a class, whereas PD standardizes this value per unit area of the landscape. Furthermore, the TE quantifies the sum of all edge lengths involving the focal class, reflecting boundary complexity. The LPI identifies the proportion of the landscape occupied by the largest patch of the class, indicating dominance. The LSI measures patch‐shape irregularity by comparing observed patch perimeters to the minimum possible perimeter for a given area, and AI quantifies the degree of spatial cohesion among patches of the same class. Collectively, these metrics provide complementary insights into patch quantity, spatial distribution, shape complexity, and aggregation for assessment of habitat configuration and potential fragmentation under current and future scenarios.

## 3 Results

### 3.1 Mitogenome Architecture

The successful sequencing and annotation of the complete mitogenome of *N. hurum* yielded a detailed understanding of its genetic makeup.

**FIGURE 3:**
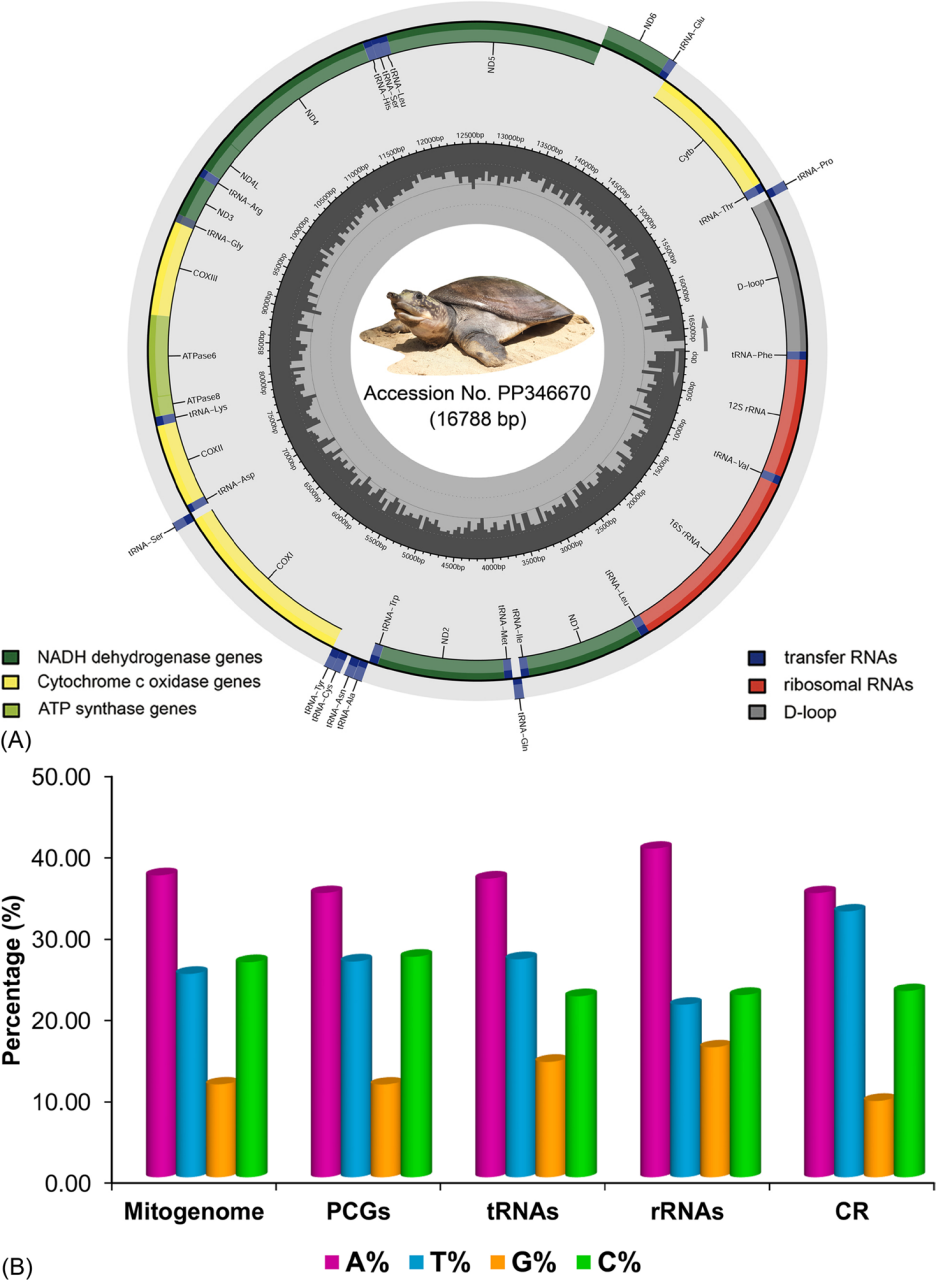
(A) The circular representation of the complete mitochondrial genome of *N. hurum* , illustrating genes encoded on the heavy and light strands. The gene categories (PCGs, rRNAs, tRNAs, and CR) are color‐coded with distinct arc boundaries. (B) The bar graph depicts the relative proportions of individual nucleotides (A, T, G, and C) in the mitogenome and within each genomic region.

The genome exhibited a circular configuration with a total length of 16,788 bp (Accession No. PP346670). The genomic structure encompassed the usual set of 37 genes with 13 PCGs, 22 tRNAs, two rRNAs, and a non‐coding control region (CR) (Figure 3A). Among them, 28 were located on the heavy strand (H‐strand), whereas the other nine genes were encoded on the light strand (L‐strand) (Table 1). The substantial variation in mitogenome lengths, ranging from 15,340 bp in *Amyda cartilaginea* to 17,499 bp in *Pelochelys cantorii* , was observed within the family Trionychidae (Table S1). Specifically, the PCGs of *N. hurum* spanned a total of 11,411 bp, accounting for 67.95% of the entire mitogenome. The rRNA genes and tRNA genes contributed 2592 bp (15.44%) and 1141 bp (6.80%) of the mitogenome, respectively, whereas the CR spanned 1288 bp, accounting for 7.67%. A total of nine intergenic spacer regions were detected, totaling 45 bp in length, with the longest spacer of 32 bp positioned between *trnN* and *trnC*. Conversely, 18 overlapping regions were detected between adjacent genes, a number twice that of the spacers, collectively spanning 118 bp. The two longest overlaps, measuring 75 bp and 10 bp, occurred between *COIII* and *trnG*, as well as between *ATP8* and *ATP6*, respectively. Most PCGs initiated with the ATG start codon, except for *COI* (GTG). The termination codon usage varied among genes: eight PCGs (*ND2*, *COII*, *ATP8*, *COIII*, *ND4L*, *ND4*, *ND5*, and *CYTB*) ended with the standard TAA stop codon, whereas *COI* and *ND6* employed the alternative stop codon AGA. Additionally, *ND1* and *ATP6* possessed incomplete stop codons represented as TA‐, and *ND3* ended with a truncated T‐‐ codon (Table 1). Furthermore, the nucleotide configuration of the *N. hurum* mitogenome revealed a marked bias toward adenine (A) and thymine (T), with contents of 37.12% and 25.01%, respectively. The proportions of A and T were 34.95% and 26.54%, whereas G and C were 11.39% and 27.11% in PCGs. The tRNA genes presented A and T contents of 36.72% and 26.82%, respectively, with G and C at 14.20% and 22.26%. Further, the rRNA genes were dominated by A (40.39%) and C (22.42%), with lower levels of T (21.22%) and G (15.97%). The CR showed the highest T content among all genome segments (32.69%) and the lowest G content (9.39%), alongside A and C proportions of 34.94% and 22.90%, respectively (Figure 3B).

**TABLE 1:** Annotated mitochondrial genes of *N. hurum* , including their genomic boundaries, gene lengths, and intergenic nucleotide regions.

Gene	Direction	Start	End	Size (bp)	Anti‐codon	Start codon	Stop codon	Intergenic nucleotide
*trnF*	+	1	70	70	GAA	—	—	−1
*12S rRNA*	+	70	1050	981	—	—	—	−3
*trnV*	+	1048	1118	71	TAC	—	—	−1
*16S rRNA*	+	1118	2728	1611	—	—	—	−1
*trnL2*	+	2728	2804	77	TAA	—	—	1
*ND1*	+	2806	3770	965	—	ATG	TA‐	−1
*trnI*	+	3770	3840	71	GAT	—	—	−2
*trnQ*	−	3839	3910	72	TTG	—	—	−2
*trnM*	+	3909	3978	70	CAT	—	—	0
*ND2*	+	3979	5019	1041	—	ATG	TAA	0
*trnW*	+	5020	5094	75	TCA	—	—	3
*trnA*	−	5098	5167	70	TGC	—	—	0
*trnN*	−	5168	5242	75	GTT	—	—	32
*trnC*	−	5275	5341	67	GCA	—	—	−1
*trnY*	−	5341	5409	69	GTA	—	—	1
*COI*	+	5411	6955	1545	—	GTG	AGA	−6
*trnS2*	−	6950	7021	72	TGA	—	—	−1
*trnD*	+	7021	7090	70	GTC	—	—	0
*COII*	+	7091	7777	687	—	ATG	TAA	0
*trnK*	+	7778	7853	76	TTT	—	—	1
*ATP8*	+	7855	8019	165	—	ATG	TAA	−10
*ATP6*	+	8010	8692	683	—	ATG	TA‐	0
*COIII*	+	8693	9547	855	—	ATG	TAA	−72
*trnG*	+	9476	9546	71	TCC	—	—	0
*ND3*	+	9547	9895	349	—	ATG	T‐‐	−1
*trnR*	+	9895	9966	72	TCG	—	—	1
*ND4L*	+	9968	10,264	297	—	ATG	TAA	−7
*ND4*	+	10,258	11,637	1380	—	ATG	TAA	0
*trnH*	+	11,638	11,708	71	GTG	—	—	−1
*trnS1*	+	11,708	11,770	63	GCT	—	—	−2
*trnL1*	+	11,769	11,841	73	TAG	—	—	0
*ND5*	+	11,842	13,620	1824	—	ATG	TAA	−5
*ND6*	−	13,616	14,140	525	—	ATG	AGA	−1
*trnE*	−	14,140	14,208	69	TTC	—	—	3
*CYTB*	+	14,212	15,351	1140	—	ATG	TAA	2
*trnT*	+	15,354	15,428	75	TGT	—	—	1
*trnP*	−	15,430	15,500	71	TGG	—	—	0
*CR*	—	15,501	16,788	1288	—	—	—	—

### 3.2 Matrilineal Phylogenetic Placement

**FIGURE 4:**
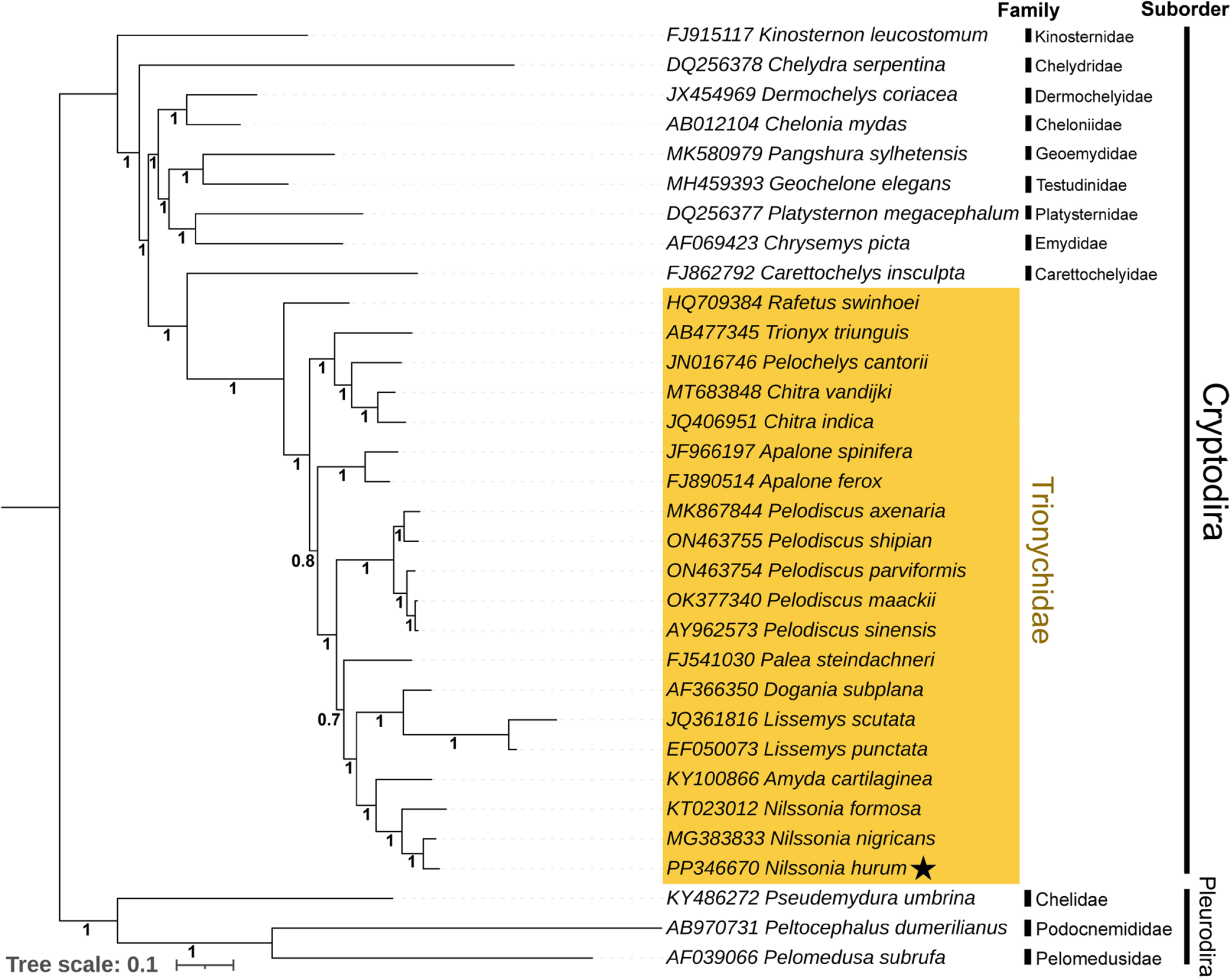
The Bayesian phylogenetic tree was inferred from concatenated sequences of 13 protein‐coding genes across representative Testudines taxa, illustrating the evolutionary placement of *N. hurum* (indicated by a black star) within the family Trionychidae. The posterior probability values of each node indicate strong support for the inferred relationships.

The mitogenome‐based phylogenetic analysis employing BI successfully resolved the evolutionary relationships among all species within the order Testudines, strongly supported by high posterior probability values at all nodes (Figure 4). Within the suborder Cryptodira, a close phylogenetic relationship was recovered among the members of the family Trionychidae and Carettochelyidae, which formed a phylogenetically distinct lineage separate from other freshwater and marine species. The analysis further revealed a cohesive monophyletic clade encompassing 20 species of Trionychidae. Notably, species within the genus *Nilssonia* formed a strongly supported monophyletic clade, with *N. hurum* showing a sister relationship with *N. nigricans* . In addition, *Rafetus swinhoei* was positioned distinctly from other trionychid taxa, suggesting it may represent a more basal or ancestral lineage within the family (Figure 4). The phylogenetic topology obtained via ML analysis was congruent with Bayesian inference results, exhibiting strong bootstrap support across all nodes, particularly within the trionychids lineage (Figure S2).

### 3.3 Genetic Distance, Haplotype Network, and Interpolated Genetic Landscape

**FIGURE 5:**
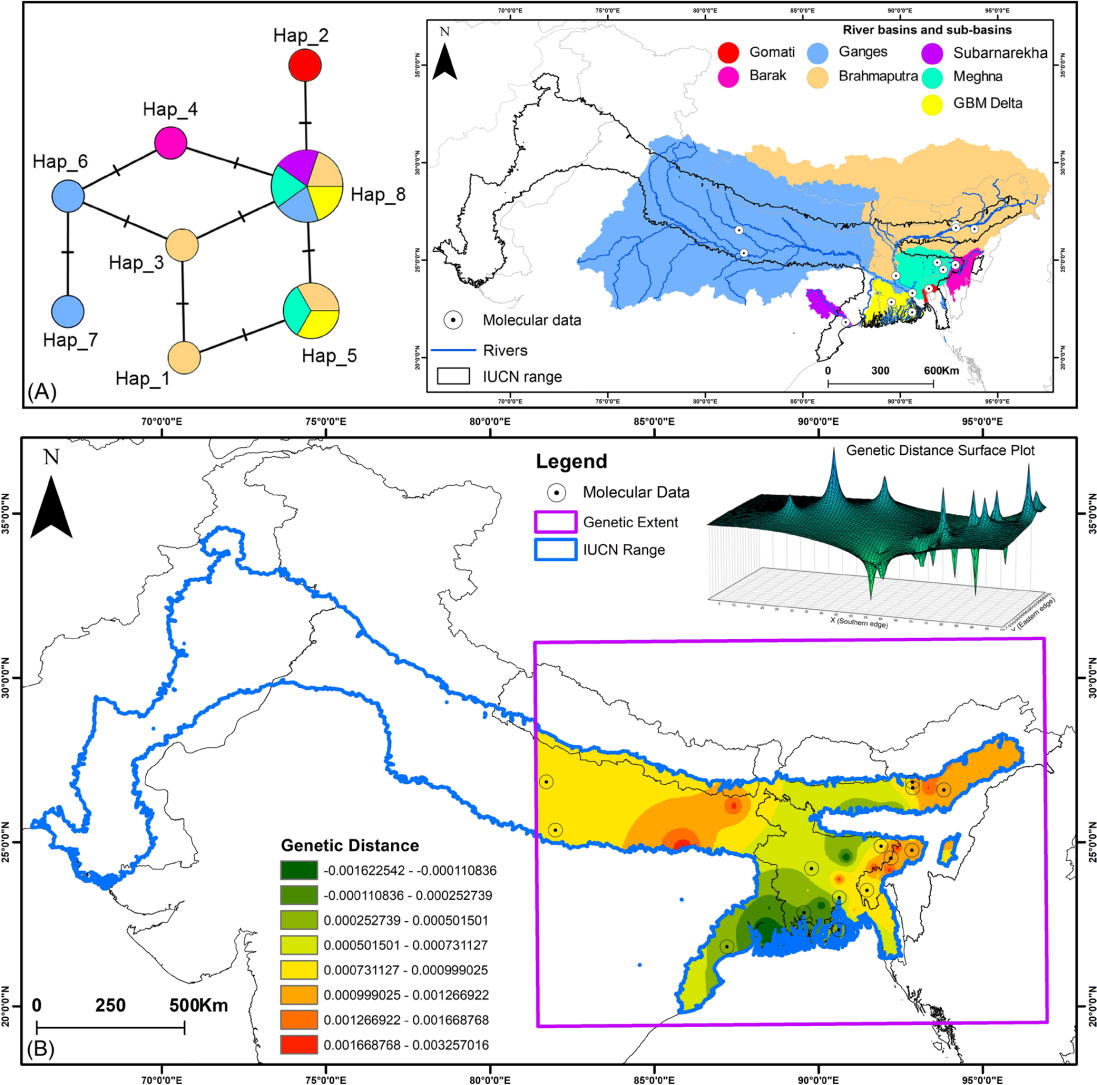
(A) TCS haplotype network constructed from the mitochondrial *CYTB* gene of *N. hurum* , showing relationships among haplotypes treated with equal frequencies. The accompanying map depicts major river basins and sub‐basins of the Indian subcontinent illustrating the geographic distribution of haplotypes. (B) The interpolated genetic distance surface plot of *N. hurum* on the basis of mitochondrial *CYTB* gene.

The intraspecific genetic distance of *N. hurum* exhibited considerable variation in the partial *CYTB* gene (0.0%–0.88%) (Table S3). The *CYTB* gene identified eight haplotypes, each characterized by four segregating sites and four parsimony‐informative sites, with Hd = 0.8091 and π = 0.37637 (Table S4). Subsequent TCS‐based haplotype network analysis revealed the presence of shared haplotypes across multiple river basins and sub‐basins (Figure 5A). The *CYTB* gene network also presented two shared haplotypes: Hap_5 was dispersed across the Meghna and Brahmaputra River basins as well as the GBM Delta, whereas Hap_8 was found in the Brahmaputra, Subarnarekha, Meghna, and Padma River basins, as well as the GBM Delta (Figure 5A). Moreover, six unique *CYTB* haplotypes were identified, each restricted to distinct river basins and sub‐basins in the Indian subcontinent. Furthermore, interpolation of pairwise genetic distances on the basis of the *CYTB* gene within the eastern range of *N. hurum* revealed a distinct genetic distance surface across multiple river basins, including the Meghna, Brahmaputra, Gomati, Ganges, Subarnarekha, Barak sub‐basin, and the GBM Delta (Figure 5B).

### 3.4 Ensemble Model Assessment and Predictor Importance

**FIGURE 6:**
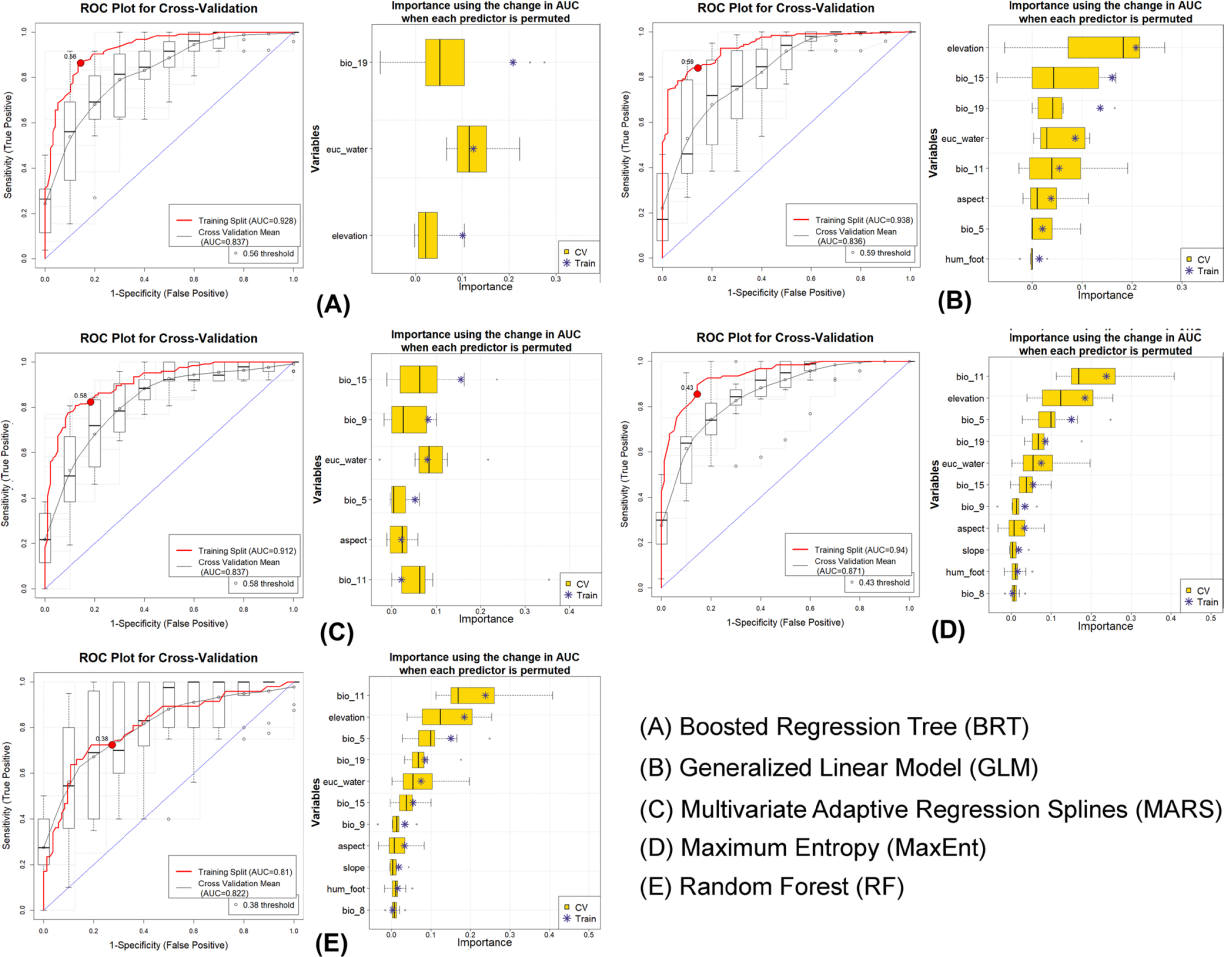
The ROC plots and variable importance analyses for five SDM algorithms applied to *N. hurum* : (A) BRT, (B) GLM, (C) MARS, (D) MaxEnt, and (E) RF. The left panels present ROC curves indicating model performance for both training and cross‐validation datasets, along with the corresponding AUC values. The right panels display the relative importance of environmental predictors as determined by each model.

The ensemble distribution model showed excellent predictive results across all five individual algorithms, with each model achieving an AUC > 0.75 in both the training and cross‐validation runs (Figure 6, Table 2). Among the algorithms, MaxEnt exhibited the highest AUC (0.94) during the training phase, whereas RF recorded the lowest training AUC (0.894). Conversely, during the cross‐validation run, the maximum AUC was observed for the RF model (0.893), whereas the GLM produced the lowest AUC (0.836). The variation between training and cross‐validation AUC values (ΔAUC) was greatest for the GLM model (0.102) and lowest for the RF model (0.001), indicating the superior generalization ability of RF compared to the other algorithms. Furthermore, additional evaluation metrics, including PCC, TSS, Kappa, sensitivity, and specificity, also demonstrated strong predictive accuracy for all models in both the training and cross‐validation datasets.

**TABLE 2:** The performance metrics of the algorithms used in the ensemble modeling for *N. hurum* .

Algorithms	Dataset	AUC	ΔAUC	PCC	TSS	Kappa	Specificity	Sensitivity
Boosted Regression Trees (BRT)	Train	0.928	0.091	86.100	0.721	0.719	0.857	0.864
	CV	0.837		78.500	0.571	0.568	0.786	0.785
Generalized Linear Models (GLM)	Train	0.938	0.102	84.800	0.697	0.693	0.857	0.84
	CV	0.836		74.600	0.483	0.484	0.696	0.787
Multivariate Adaptive Regression Splines (MARS)	Train	0.912	0.075	82.100	0.64	0.638	0.816	0.824
	CV	0.837		74.500	0.487	0.486	0.733	0.754
Maximum Entropy (MaxEnt)	Train	0.94	0.069	85.600	0.712	0.708	0.856	0.856
	CV	0.871		76.700	0.544	0.534	0.804	0.739
Random Forests (RF)	Train	0.894	0.001	85.200	0.705	0.701	0.857	0.848
	CV	0.893		83.000	0.647	0.651	0.766	0.881

Within the bioclimatic variables, Precipitation of the Coldest Quarter (bio_19) is the most influential predictor, with a mean percentage contribution of 17.91%, trailed by Precipitation Seasonality (bio_15), which contributed 15.01% (Figure 6, Table 3). Within the topographic variables, elevation was found to be the most significant predictor, accounting for 20.10% of the model's contribution. Additionally, Euclidean distance to water (euc_water) also played a critical role in elucidating habitat suitability for *N. hurum* , contributing 15.59% to the model. In comparison, the anthropogenic variable, that is, Human Influence Index (HII), showed nominal influence on the model, with a contribution of only 1.178%.

**TABLE 3:** Mean (μ) contribution of predictors to the ensemble model for *N. hurum*.

Variable	BRT	GLM	MARS	MAXENT	RF	MEAN (μ)	MEAN (μ) (%)
aspect	0.000	0.038	0.021	0.032	0.001	0.018	3.741
bio_11	0.000	0.054	0.021	0.238	0.000	0.063	12.688
bio_15	0.000	0.160	0.155	0.054	0.001	0.074	15.012
bio_19	0.208	0.136	0.000	0.084	0.014	0.088	17.908
bio_5	0.000	0.021	0.052	0.150	0.000	0.045	9.020
bio_8	0.000	0.000	0.000	0.003	0.000	0.001	0.116
bio_9	0.000	0.000	0.081	0.033	0.000	0.023	4.647
elevation	0.101	0.208	0.000	0.184	0.003	0.099	20.096
euc_water	0.124	0.086	0.080	0.074	0.022	0.077	15.593
hum_foot	0.000	0.014	0.000	0.015	0.000	0.006	1.178
slope	0.000	0.000	0.000	0.018	0.000	0.004	0.732

Abbreviations: bio_5, max temperature of the warmest month; bio_8, mean temperature of the wettest quarter; bio_9, mean temperature of the driest quarter; bio_11, mean temperature of the coldest quarter; bio_15, precipitation seasonality; bio_19, precipitation of the coldest quarter; euc_water, Euclidean distance to water; hum_foot, human influence index.

### 3.5 Habitat Suitability Assessment and Functional Connectivity

**FIGURE 7:**
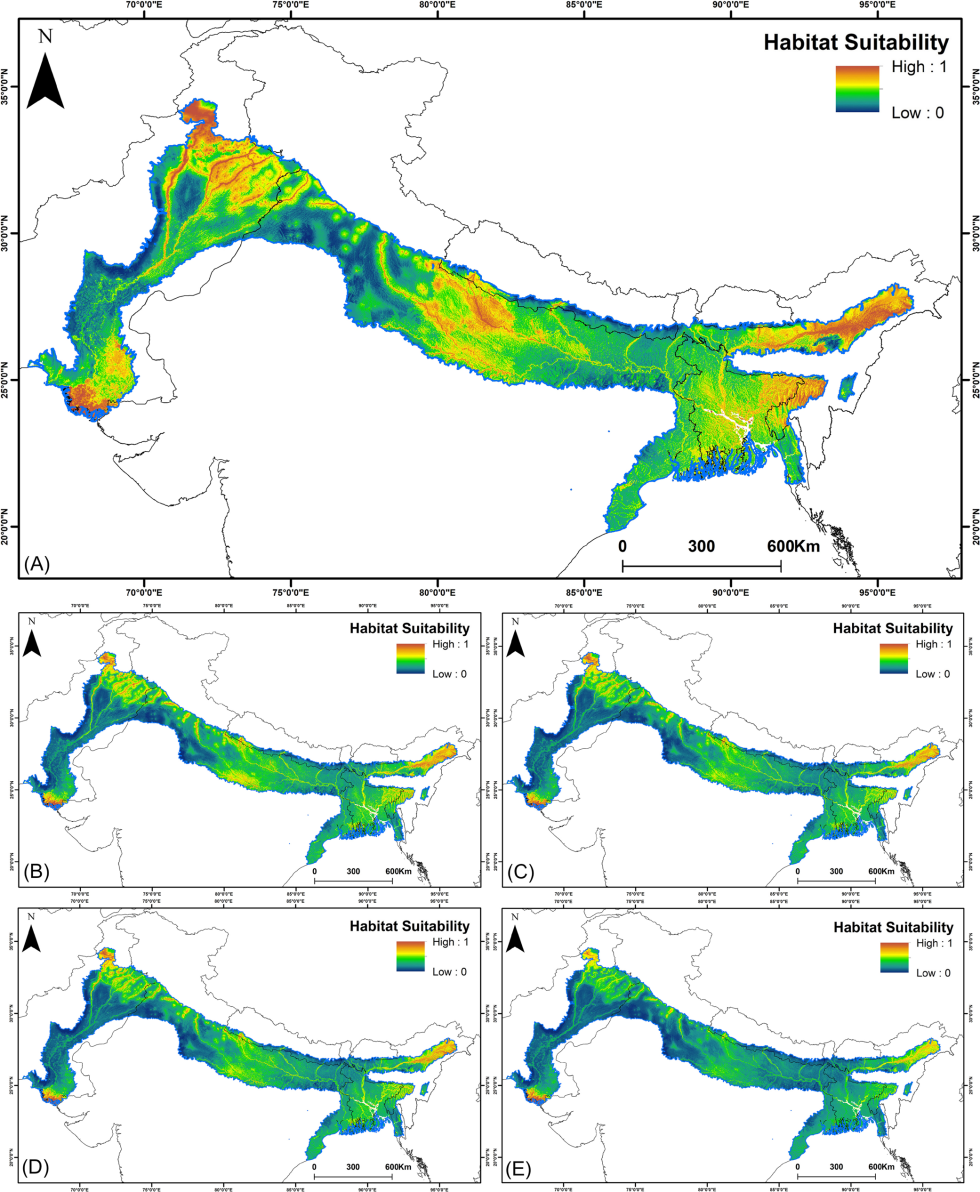
The habitat suitability of *N. hurum* in present and future climatic scenarios. (A) Present; (B) SSP245 (2041–2060); (C) SSP245 (2061–2080); (D) SSP585 (2041–2060); (E) SSP585 (2061–2080).

The ensemble model for *N. hurum* identified a total of 123,699 km^2^ of suitable habitat across the entire IUCN range under the current climatic scenario (Figure 7A, Table S5). This suitable habitat accounts for 6.81% of the total IUCN extent, which spans 1,815,920 km^2^. The future climatic projections indicate an alarming trend, as a pronounced decline is witnessed in both habitat suitability for *N. hurum* compared to the present scenario. Under the SSP245 scenario for 2041–2060, habitat suitability is projected to decrease by approximately 77.73% relative to the present (Figure 7B). This decline intensifies under the SSP245 scenario for 2061–2080, with suitable habitat areas expected to contract by 81.37% compared to the current baseline (Figure 7C). A similar, yet more severe, pattern is observed under the SSP585 scenario. During 2041–2060, habitat suitability is predicted to decline by 80.97% relative to present conditions (Figure 7D), with the most drastic reductions occurring in 2061–2080, where a decrease of 85.00% is witnessed (Figure 7E).

**FIGURE 8:**
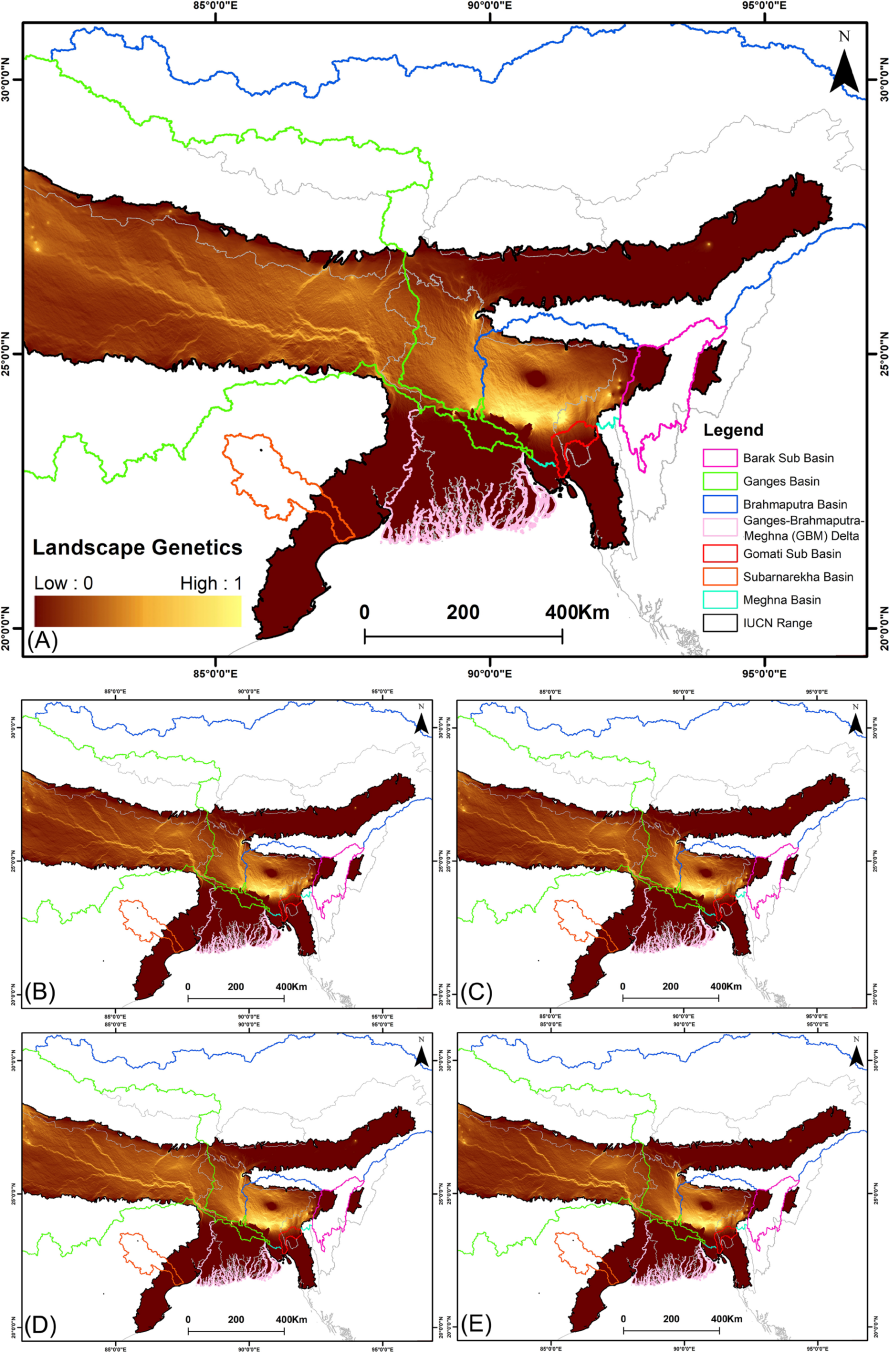
Functional connectivity of the corridor assessed through landscape genetics under present and projected future climate scenarios. (A) Present; (B) SSP245 (2041–2060); (C) SSP245 (2061–2080); (D) SSP585 (2041–2060); (E) SSP585 (2061–2080).

The landscape genetic assessment revealed distinct patterns of functional connectivity across river basins under both current and projected future scenarios (Figure 8, Table S6). In the present scenario, the Meghna basin exhibits the highest mean functional connectivity (0.603), followed by the Ganges (0.407), Gomati (0.207), and Brahmaputra basins (0.198) (Figure 8A, Table S6). In contrast, the Subarnarekha basin and the GBM delta show the lowest connectivity, with mean values of 0.001 and 0.015, respectively. The projected future scenarios, driven by anticipated losses in habitat suitability, suggest widespread declines in functional connectivity across all basins and sub‐basins (Figure 8A–8E, Table S6). Specifically, the Meghna basin is expected to experience a reduction in connectivity ranging from 5.34% to 14.97%, whereas the Ganges basin may experience losses of up to 9.04%. These reductions in connectivity are observed across all assessed basins and sub‐basins, indicating that climate‐driven habitat changes are likely to disrupt functional linkages among populations.

### 3.6 Landscape Geometry Assessment

The geometric assessment of suitable habitats reveals a critical and concerning trend under future climate scenarios for *N. hurum* . A substantial reduction in the extent of these viable patches is observed, with losses of NP ranging between 43.720% and 62.803%, primarily driven by projected declines in habitat suitability due to climate change (Table 4). This reduction in patch extent is accompanied by a notable decline in the density of the patches, as several smaller patches are entirely lost. Specifically, PD is projected to decrease by over 43.722% in future scenarios, indicating a severe loss in the number of discrete viable patches. In addition to the decline in patch numbers, the remnant patches are shrinking in size, as indicated by a reduction of 71.136% in the LPI. This shrinking of patches is further reflected in the total landscape edge, where TE decreases by 68.728%. The overall patch geometry has also become simpler, with the LSI exhibiting a decline ranging from 13.160% to 36.911%, signifying the loss of structural complexity within the remaining suitable areas. Moreover, these remnant patches have become increasingly isolated, as evidenced by the decline of AI by up to 19.839%, indicating higher fragmentation and reduced spatial connectivity. This fragmentation is expected to pose significant challenges for species dispersal and gene flow, exacerbating the vulnerability of *N. hurum* populations in the future. Overall, the geometric analysis highlights a worrying trend of fragmentation, isolation, and reduction in the size and complexity of suitable patches under future climate scenarios.

**TABLE 4:** Landscape geometry assessment of suitable patches under present and future conditions.

Scenario	NP	PD	LPI	TE	LSI	AI
Present	3511	41,247,154.17	1.3224	575.648	62.1382	78.7678
SSP245 (2041–2060)	1976	23,213,151.24	0.4594	286.64	53.9608	67.8908
SSP245 (2061–2080)	1798	21,122,088.02	0.4052	242.72	49.9013	67.5245
SSP585 (2041–2060)	1520	17,856,270.18	0.4027	202.368	46.3297	66.3823
SSP585 (2061–2080)	1306	15,342,295.3	0.3817	180.016	39.2021	63.1413

Abbreviations: AI, aggregation index; LPI, largest patch index; LSI, landscape shape index; NP, number of patches; PD, patch density; SSP, shared socioeconomic pathways; TE, total edge.

## 4 Discussion

Over the last century, extinction rates have escalated to alarming levels, with current trends indicating the onset of the sixth mass extinction event largely driven by human‐induced factors, climate change, and widespread ecological disruptions (Teixeira and Huber 2021). Consequently, the protection of biodiversity has become a pressing global priority, essential for maintaining ecological balance and supporting human welfare. Addressing this challenge requires the establishment of cohesive scientific frameworks and the execution of comprehensive conservation measures (Conde et al. 2019). Reptiles, accounting for nearly one‐third of all tetrapod diversity worldwide, remain one of the most underrepresented groups in conservation initiatives, despite their significant ecological role. Approximately 20% of reptilian species among them are currently considered threatened and facing extinction risks (Cox et al. 2022). The lack of attention to this group highlights the urgent need for interdisciplinary approaches that integrate genetic, ecological, and spatial data to guide conservation priorities for these cold‐blooded vertebrates (Meiri et al. 2023). Thus, addressing modern biodiversity crises increasingly depends on integrative assessment methods, which combine multiple biological and environmental datasets to develop effective conservation strategies (McMahon et al. 2011). These approaches have been instrumental in guiding conservation decisions and reducing extinction risks for numerous species (Gutiérrez‐Rodríguez et al. 2017). Such frameworks are also strongly emphasized within global conservation agreements, particularly under the Convention on Biological Diversity, which promotes advanced, evidence‐based management strategies to mitigate biodiversity loss (CBD 2010; Hoban et al. 2021; CBD 2022). This field of landscape genetics has emerged as a synergistic discipline that combines principles and methodologies from population genetics, landscape ecology, and geography, which has proven effective in mitigating biodiversity loss by formulating robust conservation strategies (Storfer et al. 2010). In alignment with global conservation priorities, the present study elucidates the mitogenomic data of *N. hurum* and integrates this information with habitat suitability to characterize the genetic landscape within its current range in present and future climatic scenarios. This integrative approach establishes a scientifically robust foundation for the formulation of targeted conservation strategies and long‐term management plans for this threatened freshwater turtle species (Gallego‐García et al. 2021).

In this study, the novel mitogenome of *N. hurum* exhibits an asymmetric gene distribution, with 28 genes encoded on the heavy strand and nine on the light strand, reflecting strand‐specific replication and transcriptional regulation consistent with patterns observed in other *Nilssonia* species (Kundu et al. 2018). Specifically, the current findings corroborate earlier studies using mitochondrial and nuclear DNA data that confirm the monophyly of the family Trionychidae and the close phylogenetic relationship between *N. hurum* and *N. nigricans* (Le et al. 2014; Kundu et al. 2018). The mitogenome‐based phylogenetic analysis presented here provides more comprehensive evidence and higher resolution for affirming the systematics of *N. hurum* and matrilineal evolutionary relationships among trionychids. Moreover, the intraspecific genetic distance, haplotype, and nucleotide diversity in partial *CYTB* sequences indicate notable genetic variation among populations associated with different river basins, consistent with patterns reported in other freshwater turtles where geographic barriers such as river systems shape population structure (Chaianunporn et al. 2024; Young et al. 2025). However, the presence of shared haplotypes across distinct river basins and sub‐basins indicates either historical or ongoing gene flow among *N. hurum* populations or human‐mediated translocation, a pattern commonly observed in turtles inhabiting hydrologically interconnected freshwater systems. However, the presence of shared haplotypes across distinct river basins and sub‐basins suggests either historical or ongoing gene flow among *N. hurum* populations, a pattern commonly reported in turtles inhabiting hydrologically interconnected freshwater systems, or alternatively human‐mediated translocation (Todd et al. 2014; Agostini et al. 2024). Conversely, the presence of exclusive haplotypes constrained to specific localities suggests localized genetic differentiation, likely driven by microhabitat selection and limited dispersal capacity (Toha et al. 2025).

The ensemble SDM indicated that suitable areas across the entire IUCN range of *N. hurum* are relatively low, as only 123,699 km^2^ were identified as suitable under current conditions, which accounted for 6.81% of the total range. Notably, the identified suitable areas are primarily influenced by temperature‐ and precipitation‐related bioclimatic variables, as well as the availability of wetlands. The bioclimatic variables such as Precipitation of the Coldest Quarter (bio_19), Precipitation Seasonality (bio_15), and Mean Temperature of the Coldest Quarter (bio_11) with mean percentage contribution of 17.908%, 15.012%, and 12.688% respectively, played a pivotal role in the ectothermic physiology of this trionychid species (Santoro et al. 2023). These climatic factors are fundamental for regulating hydration, foraging activity, and other vital biological processes, as highlighted in previous studies (Butler 2019). The results align with existing studies, which found that environmental parameters significantly impact the distribution and ecological dynamics of reptilian species (Biber et al. 2023). The temperature critically influences skewed sex ratios by affecting the expression of sex‐determining genes in many reptilian species, including freshwater turtles (Santoro et al. 2023). The precipitation regimes are expected to significantly influence hydrological dynamics and can directly influence the accessibility, stability, and quality of aquatic regimes that are crucial for juvenile persistence and growth (Geller et al. 2022). Alterations in precipitation patterns may lead to habitat desiccation during prolonged dry periods or excessive flooding during extreme rainfall events, both of which can disrupt resource availability, alter foraging opportunities, and reduce access to safe refuges from predators (Santoro et al. 2024). Additionally, Euclidean distance to water bodies, influencing 15.59% of the habitat suitability model, was recognized as another key determinant. This highlights the need to protect riparian areas and nearby river habitats, as they can play a crucial role in supporting healthy populations of freshwater turtles, including *N. hurum* (Buhlmann et al. 2009; Willey et al. 2022). Collectively, these climatic pressures have the potential to reduce reproductive success, hinder juvenile development, and disrupt population connectivity, thereby threatening the species' long‐term survival. Consequently, the areas identified as potential climate refugia become crucial zones for sustaining populations under both current and projected future climate change scenarios (Durance and Ormerod 2007).

However, the future projections indicate highly concerning scenarios, as habitat suitability for *N. hurum* is predicted to decline drastically. These declines exceeding 77% within the IUCN range compared to current conditions are primarily driven by climate‐induced shifts. Such alarming trends of habitat loss due to changing climatic regimes have been well‐documented in recent ecological studies (Mothes et al. 2020; Willey et al. 2022). The projected reduction in suitable habitat underscores the increasing risk that environmental conditions may become insufficient to support the persistence of this trionychid turtle in many parts of its range (Santoro et al. 2023; Gregory et al. 2024). The regions, including the Northern and Southern Indus, Central Ganges, Eastern Brahmaputra, and the central part of the Meghna River Basin, which are identified as suitable patches, represent critical areas of conservation significance. However, the projected contraction of suitable regions is expected not only to reduce the geographic distribution of *N. hurum* but also to trigger cascading effects on broader ecosystem functioning and interspecific interactions (Janzen et al. 2018; Piczak et al. 2025). This situation is further exacerbated by existing anthropogenic pressures, with climate change compounding the problem through severe fragmentation and alteration of the remaining habitat patches (Stanford et al. 2020; Fischer et al. 2021). The reduction of multiple suitable patches, as shown by decreasing values of NP and PD, illustrates this trend. The remnant patches are becoming smaller, more fragmented, and increasingly isolated, as reflected by substantial reductions in key landscape metrics such as the LPI, TE, and AI. These structural changes highlight an accelerating fragmentation process within the species' current distributional range. Additionally, these Himalayan‐origin riverine systems and their associated biota have already been demonstrated to exhibit elevated susceptibility to environmental stressors, particularly those arising from climate change and escalating anthropogenic pressures (Uereyen et al. 2022). Hence, this fragmentation is expected to severely impair landscape connectivity, thereby constraining dispersal pathways and reducing genetic exchange among different populations.

The landscape genetics analysis revealed substantial variation in functional connectivity among subpopulations, broadly consistent with patterns of genetic differentiation across basins and sub‐basins. The Meghna basin exhibited the highest functional connectivity, reflecting strong gene flow and correspondingly low genetic distances among populations. In the Ganges basin, connectivity was moderate, matching intermediate levels of genetic differentiation. However, the southern portion of the Ganges basin showed reduced connectivity and elevated genetic distances, likely influenced by factors such as siltation or the presence of dams in upstream regions. In contrast, the Brahmaputra basin and the Barak sub‐basin also showed very low functional connectivity despite having suitable patches. This pattern also corresponds with the observation that these regions exhibit the highest genetic distances among populations. The reduced connectivity may be driven by seasonal fluctuations in river levels, the formation of isolated oxbow lakes disconnected from the main river channel, and the prevalence of isolated ponds in temple areas or other human‐modified landscapes. The GBM Delta, Gomati, and Subarnarekha basins, despite having low genetic distances, displayed low connectivity that may reflect the scarcity of suitable habitat in these basins. Moreover, the sparse and scattered occurrence records further suggest that populations are likely small, fragmented, or stray. Notably, future projections indicate a decline in functional connectivity across nearly all basins and sub‐basins, emphasizing the potential impacts of climate‐driven habitat changes on population persistence. Overall, connectivity is shaped by a combination of factors such as habitat suitability, geographic and topographic barriers, fluctuating water levels, and siltation, all of which can restrict dispersal. In light of these factors, site‐specific field assessments within suitable habitats and identified functional corridors are recommended to evaluate local barriers and guide targeted conservation measures. Moreover, the populations inhabiting these regions may serve as vital source stocks for translocation initiatives aimed at establishing new populations in climatically favorable habitats. Additionally, they are of high value for ex situ conservation and captive breeding programs to safeguard genetic diversity (Forsman 2014; Kardos et al. 2021). Hence, this combined approach, therefore, provided a more realistic understanding of functional connectivity than habitat suitability alone, linking ecological suitability to underlying genetic structure.

## 5 Conservation Implications

The present study establishes a foundational reference for mitogenomic and ecological datasets, supporting landscape genetics and conservation planning for *N. hurum* in South Asia. Nevertheless, as the existing genetic data for *N. hurum* are primarily derived from major river basins and sub‐basins in the eastern regions of the Indian subcontinent, it is strongly recommended to generate comparable datasets from the Indus River Basin to achieve a more comprehensive understanding. On the basis of the findings of this study, suitable habitat patches with high genetic distances and low functional connectivity, particularly in the Central Ganges, Eastern Brahmaputra, and Central Meghna River basins, should be prioritized as conservation units for targeted conservation actions. These areas warrant focused efforts to identify viable populations of *N. hurum* that may serve as individuals for future translocation or reintroduction programs. Additionally, enhancing connectivity among the fragmented and isolated populations and the suitable habitats identified in this study is equally important to facilitate natural gene flow. This prioritization should be particularly directed toward the central part of the Ganges and Meghna River Basins, where projected losses are notably more severe compared to other regions within the species' range. This should begin with detailed field assessments at specific sites within these regions to better understand local barriers and to facilitate site‐specific interventions aimed at improving connectivity. Moreover, particular attention should be directed toward the Meghna basin, where severe losses of habitat and functional connectivity have been observed. Addressing these issues is critical, as they may pose significant challenges to gene flow in the region in the future. Such measures are vital not only to maintain genetic diversity but also to minimize the risk of inbreeding depression. Furthermore, proactive and site‐specific management actions should be implemented to address key anthropogenic pressures within the prioritized areas identified by this research. Certain actions include the strict regulation and control of sand/silt mining, stone quarrying, and illegal fishing, particularly those concerning nylon gill nets and electric shock fishing. The protection and restoration of sandbars, riparian zones, and adjacent floodplain habitats must be prioritized, as these zones provide critical nesting and breeding grounds for *N. hurum* and other freshwater turtle species. Moreover, heightened surveillance and community‐based conservation programs are necessary to curb egg collection, illegal poaching of hatchlings for the pet trade, and manage predation threats posed by stray local dogs and invasive predators. Additionally, amusement events such as tourism and picnicking on sandbars should be attentively monitored and controlled to prevent habitat disturbance during the sensitive breeding and nesting periods. Any proposed developmental or infrastructural projects within these naturally fragile habitats need to undergo rigorous environmental impact assessments to ensure that such activities align with established conservation objectives. In the broader context of conservation, management strategies for *N. hurum* should also incorporate other closely related congeners under the same conservation framework, ensuring a holistic and ecosystem‐level approach to freshwater turtle conservation. Lastly, fine‐scale population genetic studies, in combination with ecological niche modeling and landscape genomic analyses, are strongly recommended within or beyond the current distribution range of *N. hurum* . This is particularly important for the western part of the species range in the Indus basin, where genetic data are currently lacking. Incorporating these areas in future landscape genetics studies is therefore recommended to gain a comprehensive understanding of connectivity and gene flow for the species. Such integrated assessments will provide essential insights for supporting the formulation of robust, evidence‐based strategies for the long‐term conservation of this imperiled testudine species.

## 6 Limitations

The present study has some inherent limitations that should be addressed in future research. It relied on a single mitochondrial gene, and future studies could benefit from incorporating additional genetic data across the entire range of the species, particularly within the Indus Basin and Upper Ganges. Molecular data from mitochondrial, nuclear, and single nucleotide polymorphism markers will help provide a more accurate assessment of the population structure of *N. hurum* , whereas whole‐genome data will further refine its systematics. Thus, expanding genetic data would enable more comprehensive landscape genetic analyses, as the current study was limited to a partial gene fragment and constrained by available data from the majority of the river basins. Additionally, the study utilized only a single General Circulation Model (GCM) for climatic projections, and future research should incorporate multiple GCMs to improve the robustness and reliability of distribution predictions. Moreover, field‐based assessments of species presence in suitable zones are recommended to refine future distribution modeling. Understanding potential barriers in different river basins, where connectivity was found to be low in this study, is also crucial. Overall, this study provides a foundational framework, and future research building on these findings can contribute to more effective conservation planning and management strategies for the species.

## 7 Conclusion

The integrative conservation approach, combining both genetic and ecological tools, has proven effective in guiding proactive strategies for the protection of numerous threatened species worldwide. Despite being among the most ancient and imperiled vertebrates, freshwater turtles such as *N. hurum* have remained largely underrepresented in multidisciplinary conservation frameworks. In this study, an integrative approach incorporating mitochondrial genomics and ensemble habitat modeling was employed to assess the landscape genetics of *N. hurum* and inform effective management strategies. The complete mitogenome of *N. hurum* provided critical insights into its genomic architecture and organization, whereas phylogenetic analysis confirmed a close matrilineal relationship with *N. nigricans* . The genetic distance estimates and haplotype network analyses indicated substantial intraspecific diversity and the occurrence of shared haplotypes across multiple river basins and sub‐basins in the eastern regions of the Indian subcontinent. The ensemble species distribution models, integrated with genetic surfaces on the basis of multiple mitochondrial markers, identified climatically suitable habitats and functional corridor connectivity within the IUCN range. However, these areas are projected to decline significantly and become increasingly fragmented under future climate scenarios. Therefore, this integrated landscape genetics approach provides a strong scientific basis for informed conservation strategies targeting *N. hurum* . Such efforts will enhance long‐term population sustainability by mitigating inbreeding depression and lowering the risk of further population declines in multiple freshwater turtle species across South Asia.

## Author Contributions

**Imon Abedin:** data curation (supporting), formal analysis (equal), methodology (supporting), software (equal), writing – original draft (supporting). **Angkasa Putra:** data curation (supporting), formal analysis (equal), methodology (supporting), software (equal), writing – original draft (supporting). **Hye‐Eun Kang:** investigation (equal), writing – original draft (supporting). **Arunima Singh:** data curation (supporting), investigation (equal), methodology (supporting). **Shailendra Singh:** validation (equal), visualization (equal), writing – review and editing (supporting). **Hilloljyoti Singha:** validation (equal), visualization (equal). **Hyun‐Woo Kim:** resources (equal), supervision (equal), writing – review and editing (supporting). **Shantanu Kundu:** conceptualization (lead), funding acquisition (lead), project administration (lead), resources (equal), supervision (equal), writing – review and editing (supporting).

## Funding

This research was supported by the Autonomous Creative Academic Research Fund (202416560001), Pukyong National University 2024–2025, granted to S.K.

## Conflicts of Interest

The authors declare no conflicts of interest.

## Supporting information


**Data S1:** ece372751‐sup‐0001‐DataS1.docx.

## Data Availability

The assembled mitochondrial genome has been submitted to the GenBank database (https://www.ncbi.nlm.nih.gov/) under accession number PP346670. All datasets utilized in the analyses were obtained from publicly accessible sources. Species occurrence data were extracted using the Geospatial Conservation Assessment Tool (GeoCAT) (https://geocat.iucnredlist.org/). Bioclimatic variables were sourced from the WorldClim database (https://www.worldclim.org/), and topographic data were derived from NASA's Shuttle Radar Topography Mission (SRTM) dataset (http://srtm.csi.cgiar.org/srtmdata/). Habitat and anthropogenic variables were retrieved from land use and land cover (LULC) datasets provided by the Copernicus Global Land Service (https://land.copernicus.eu/). Outputs from ecological modeling and genetic analyses are presented in the main text and Supporting Information. For further data inquiries, please contact the corresponding author (S.K.).
