# Supplementary material for: Cladistic Relationships and Landscape Genetics of the Endangered Indian Peacock Softshell Turtle Nilssonia hurum (Gray, 1830): Implications for Strategic Conservation Planning
Source: Ecol Evol. 2025 Dec 22;15(12):e72751. doi: 10.1002/ece3.72751 (PMC12720016; doi:10.1002/ece3.72751)
Supplement: Supplementary file 1 — Data S1: ece372751‐sup‐0001‐DataS1.docx. [file ECE3-15-e72751-s001.docx]

**SUPPORTING INFORMATION**

**Cladistic Relationships and Landscape Genetics of the Endangered Indian Peacock Softshell Turtle *Nilssonia hurum* (Gray, 1830): Implications for Strategic Conservation Planning**

Imon Abedin^1^, Angkasa Putra^2^, Hye-Eun Kang^3^, Arunima Singh^4^, Shailendra Singh^4^, Hilloljyoti Singha^1,5^, Hyun-Woo Kim^6,7,8^, Shantanu Kundu^2,9,10^*

^1^ Wildlife Ecology Lab, Department of Zoology, Bodoland University, Kokrajhar 783370, Assam, India

^2^ Interdisciplinary Program of Marine and Fisheries Sciences and Convergent Technology, Pukyong National University, Busan 48513, Republic of Korea

^3^ Institute of Marine Life Science, Pukyong National University, Busan 48513, Republic of Korea.

^4^ Turtle Survival Alliance Foundation India, Lucknow 226 012, India.

^5^ Centre for Wildlife Research and Biodiversity Conservation, Bodoland University, Kokrajhar 783370, Assam, India

^6^ Department of Marine Biology, College of Fisheries Science, Pukyong National University, Busan 48513, Republic of Korea.

^7^ Research Center for Marine Integrated Bionics Technology, Pukyong National University, Busan 48513, Republic of Korea

^8^ Marine Integrated Biomedical Technology Center, National Key Research Institutes in Universities, Pukyong National University, Busan 48513, Republic of Korea

^9^ Ocean and Fisheries Development International Cooperation Institute, College of Fisheries Science, Pukyong National University, Busan 48513, Republic of Korea.

^10^ International Graduate Program of Fisheries Science, Pukyong National University, Busan 48513, Republic of Korea.

*Corresponding author at: Ocean and Fisheries Development International Cooperation Institute, College of Fisheries Science, Pukyong National University, Busan 48513, Republic of Korea. [shantanu1984@pknu.ac.kr](mailto:shantanu1984@pknu.ac.kr); shantanu1984@gmail.com (S. Kundu).

**Table S1.** Details of mitochondrial genome sequences used in this study, including newly generated data and sequences retrieved from the GenBank database, along with corresponding accession numbers and genome length.

| **No.** | **Sub-Order** | **Family** | **Species Name** | **Accession No.** | **Size (bp)** |
| --- | --- | --- | --- | --- | --- |
| 1 | Cryptodira | Trionychidae | *Nilssonia hurum* | PP346670 | 16,788 |
| 2 | Cryptodira | Trionychidae | *Nilssonia formosa* | KT023012 | 17,149 |
| 3 | Cryptodira | Trionychidae | *Nilssonia nigricans* | MG383833 | 16,796 |
| 4 | Cryptodira | Trionychidae | *Amyda cartilaginea* | KY100866 | 15,340 |
| 5 | Cryptodira | Trionychidae | *Apalone ferox* | FJ890514 | 16,866 |
| 6 | Cryptodira | Trionychidae | *Apalone spinifera* | JF966197 | 16,749 |
| 7 | Cryptodira | Trionychidae | *Chitra indica* | JQ406951 | 16,726 |
| 8 | Cryptodira | Trionychidae | *Chitra vandijki* | MT683848 | 16,614 |
| 9 | Cryptodira | Trionychidae | *Dogania subplana* | AF366350 | 17,289 |
| 10 | Cryptodira | Trionychidae | *Lissemys punctata* | EF050073 | 16,490 |
| 11 | Cryptodira | Trionychidae | *Lissemys scutata* | JQ361816 | 16,512 |
| 12 | Cryptodira | Trionychidae | *Palea steindachneri* | FJ541030 | 17,243 |
| 13 | Cryptodira | Trionychidae | *Pelochelys cantorii* | JN016746 | 17,499 |
| 14 | Cryptodira | Trionychidae | *Pelodiscus axenaria* | MK867844 | 16,593 |
| 15 | Cryptodira | Trionychidae | *Pelodiscus maackii* | OK377340 | 16,258 |
| 16 | Cryptodira | Trionychidae | *Pelodiscus parviformis* | ON463754 | 17,095 |
| 17 | Cryptodira | Trionychidae | *Pelodiscus shipian* | ON463755 | 17,300 |
| 18 | Cryptodira | Trionychidae | *Pelodiscus sinensis* | AY962573 | 17,042 |
| 19 | Cryptodira | Trionychidae | *Rafetus swinhoei* | HQ709384 | 16,990 |
| 20 | Cryptodira | Trionychidae | *Trionyx triunguis* | AB477345 | 16,590 |
| 21 | Cryptodira | Testudinidae | *Geochelone elegans* | MH459393 | 16,446 |
| 22 | Cryptodira | Geoemydidae | *Pangshura sylhetensis* | MK580979 | 16,568 |
| 23 | Cryptodira | Emydidae | *Chrysemys picta* | AF069423 | 16,866 |
| 24 | Cryptodira | Dermochelyidae | *Dermochelys coriacea* | JX454969 | 16,420 |
| 25 | Cryptodira | Cheloniidae | *Chelonia mydas* | AB012104 | 16,497 |
| 26 | Cryptodira | Platysternidae | *Platysternon megacephalum* | DQ256377 | 19,043 |
| 27 | Cryptodira | Kinosternidae | *Kinosternon leucostomun* | FJ915117 | 16,559 |
| 28 | Cryptodira | Chelydridae | *Chelydra serpentina* | DQ256378 | 14,572 |
| 29 | Cryptodira | Carettochelyidae | *Carettochelys insculpta* | FJ862792 | 16,439 |
| 30 | Pleurodira | Chelidae | *Pseudemydura umbrina* | KY486272 | 16,414 |
| 31 | Pleurodira | Podocnemididae | *Peltocephalus dumerilianus* | AB970731 | 16,601 |
| 32 | Pleurodira | Pelomedusidae | *Pelomedusa subrufa* | AF039066 | 16,787 |

**Table S2. Details of the partial mitochondrial *CYTB* genes employed for estimating genetic distances and constructing haplotype networks. The sequence generated by this study is marked with asterisk (*).**

| **Accession No.** | **River Basin** |
| --- | --- |
| PP346670***** | Ganges |
| OP617709 | Brahmaputra |
| OP617708 | Gomati |
| OP617707 | Brahmaputra |
| OP617706 | Barak |
| OP617705 | Brahmaputra |
| MT939578 | Ganges |
| MT939577 | Ganges |
| MT939576 | Ganges |
| MT939575 | Ganges |
| MT939574 | Ganges |
| MF432961 | Ganges |
| MF432960 | Ganges |
| HE801756 | Subarnarekha |
| HE801755 | Meghna |
| AY259548 | Ganges |
| HE801754 | Meghna |
| HE801753 | GBM Delta system |
| HE801752 | GBM Delta system |
| JN232528 | Brahmaputra |
| AM495224 | Brahmaputra |
| AM495223 | Meghna |
| AM495222 | Meghna |
| AM495221 | Brahmaputra |
| AM495220 | Brahmaputra |
| AM495219 | Ganges |
| AM495218 | Ganges |

**Table S3.** Genetic distance and diversity *N. hurum* based on the partial mitochondrial *CYTB* genes.

| **Parameter** | ***CYTB*** |
| --- | --- |
| **Genetic distance range** |  |
| Intra-species | 0.0–0.88 |
| **Genetic diversity indices** |  |
| Number of sequences (N) | 27 |
| Number of polymorphic sites (P) | 4 |
| Number of haplotypes (H) | 8 |
| Average number of nucleotide differences (K) | 1.54986 |
| Haplotype diversity (Hd) | 0.80910 |
| Nucleotide diversity (π) | 0.37637 |

**Table S4.** The sequences of the partial mitochondrial *CYTB* genes were grouped according to distinct estimated haplotypes.

| **Haplotype** | **n** | **Sequences** |
| --- | --- | --- |
| ***CYTB*** | | |
| Hap_1 | 1 | OP617709 |
| Hap_2 | 1 | OP617708 |
| Hap_3 | 1 | OP617707 |
| Hap_4 | 1 | OP617706 |
| Hap_5 | 6 | OP617705, HE801755, HE801754, HE801752, AM495221, AM495220 |
| Hap_6 | 6 | MT939578, MT939577, MT939576, MT939574, MF432960, PP346670 |
| Hap_7 | 2 | MT939575, MF432961 |
| Hap_8 | 9 | HE801756, AY259548, HE801753, JN232528, AM495224, AM495223, AM495222, AM495219, AM495218 |

**Table S5.** The area (in km²) of habitat suitability under present and future climatic scenarios.

| **Scenario** | **Area** |
| --- | --- |
| Present | 123699 |
| SSP245 (2041-2060) | 27546 |
| SSP245 (2061-2080) | 23040 |
| SSP585 (2041-2060) | 23542 |
| SSP585 (2061-2080) | 18554 |

**Table S6.** The mean functional corridor connectivity within different basins and sub basins delineated through landscape genetics in present and future scenarios.

| **Basin/Sub basin** | **Present** | **SSP245 (2041-2060)** | **SSP245 (2061-2080)** | **SSP585 (2041-2060)** | **SSP585 (2061-2080)** |
| --- | --- | --- | --- | --- | --- |
| Meghna | 0.603 | 0.571 | 0.549 | 0.528 | 0.513 |
| Ganges | 0.407 | 0.400 | 0.393 | 0.380 | 0.370 |
| Gomati | 0.207 | 0.203 | 0.204 | 0.207 | 0.207 |
| Brahmaputra | 0.198 | 0.190 | 0.190 | 0.190 | 0.190 |
| Barak | 0.052 | 0.050 | 0.050 | 0.050 | 0.050 |
| GBM Delta | 0.015 | 0.015 | 0.015 | 0.015 | 0.015 |
| Subarnarekha | 0.001 | 0.001 | 0.001 | 0.001 | 0.001 |





**Figure S1.** Correlation matrix (threshold < 0.7) among covariates selected for the final model of *N. hurum*. Pearson correlation coefficients are primarily used. In cases where the Spearman or Kendall coefficients are higher than the Pearson's, the corresponding box includes an "s" or "k" in the lower right corner to indicate this.


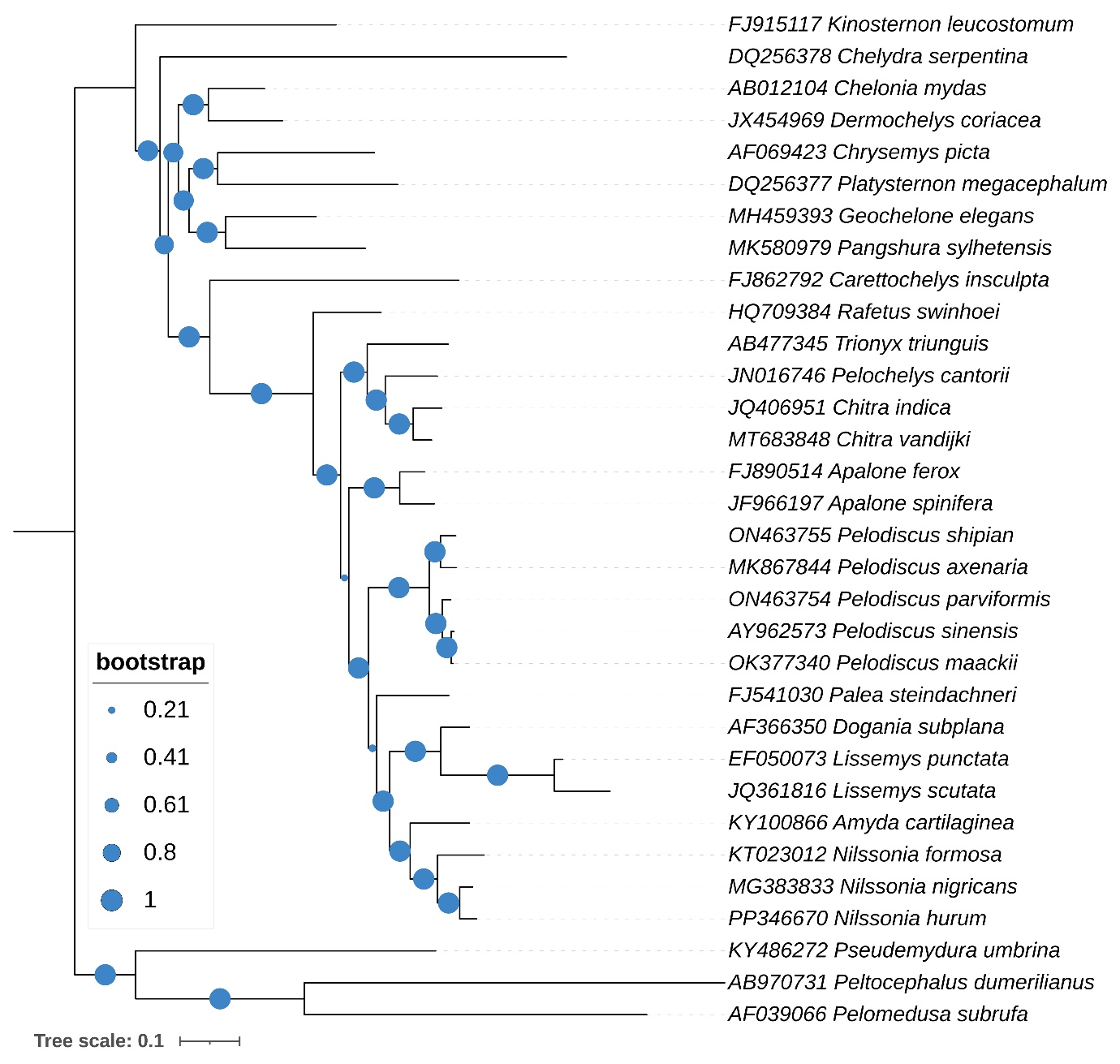


**Figure S2.** Maximum-Likelihood (ML) phylogenetic tree depicting the evolutionary relationships between *N. hurum* and other Testudines species. Bootstrap support is visualized using blue circles of different sizes placed at each node, reflecting support strength.
